# Association of Common Variants in MMPs with Periodontitis Risk

**DOI:** 10.1155/2016/1545974

**Published:** 2016-04-19

**Authors:** Wenyang Li, Ying Zhu, Pradeep Singh, Deepal Haresh Ajmera, Jinlin Song, Ping Ji

**Affiliations:** ^1^Chongqing Key Laboratory of Oral Diseases and Biomedical Sciences and College of Stomatology, Chongqing Medical University, Chongqing 400016, China; ^2^Department of Forensic Medicine, Faculty of Basic Medical Sciences, Chongqing Medical University, Chongqing 400016, China

## Abstract

*Background.* Matrix metalloproteinases (MMPs) are considered to play an important role during tissue remodeling and extracellular matrix degradation. And functional polymorphisms in MMPs genes have been reported to be associated with the increased risk of periodontitis. Recently, many studies have investigated the association between MMPs polymorphisms and periodontitis risk. However, the results remain inconclusive. In order to quantify the influence of MMPs polymorphisms on the susceptibility to periodontitis, we performed a meta-analysis and systematic review.* Results.* Overall, this comprehensive meta-analysis included a total of 17 related studies, including 2399 cases and 2002 healthy control subjects. Our results revealed that although studies of the association between* MMP-8* −799 C/T variant and the susceptibility to periodontitis have not yielded consistent results,* MMP-1* (−1607 1G/2G, −519 A/G, and −422 A/T),* MMP-2* (−1575 G/A, −1306 C/T, −790 T/G, and −735 C/T),* MMP-3* (−1171 5A/6A),* MMP-8* (−381 A/G and +17 C/G),* MMP-9* (−1562 C/T and +279 R/Q), and* MMP-12* (−357 Asn/Ser), as well as* MMP-13* (−77 A/G, 11A/12A) SNPs are not related to periodontitis risk.* Conclusions.* No association of these common MMPs variants with the susceptibility to periodontitis was found; however, further larger-scale and multiethnic genetic studies on this topic are expected to be conducted to validate our results.

## 1. Introduction

Periodontitis being one of the most common forms of destructive periodontal disease in adults can be defined as bacterial plaque induced inflammation of the attachment apparatus of teeth and supporting structures, which initially manifests as gingivitis and is characterized by extension of inflammation from the gingiva into deeper periodontal tissues that if left untreated results in destruction of periodontium associated with progressive attachment loss and irreversible bone loss [1]. Currently, periodontitis is considered to be multifactorial disease, developing as a result of complex interactions between specific host genes and the environment [2]. Although periodontitis is initiated and sustained by bacterial plaque, host factors determine the pathogenesis and rate of progression of the disease [3].

Matrix metalloproteinases (MMPs) are a large family of metal-dependent extracellular proteinases which are responsible for the tissue remodeling and degradation of the extracellular matrix (ECM), including collagens, elastins, gelatin, matrix glycoproteins, and proteoglycans [4]. To date, at least 26 members of MMPs have been identified [5]. The majority of MMPs proteins are secreted as inactive proMMPs, which are subsequently processed by other proteolytic enzymes (such as serine proteases, furin, and plasmin) to generate the active forms. The proteolytic activities of MMPs are precisely controlled during activation from their precursors and inhibition by endogenous inhibitors, a-macroglobulins, and tissue inhibitors of metalloproteinases (TIMPs) or by nonselective synthetic inhibitors (batimastat, BB-94) [6].

Significant evidence suggests that MMPs comprise the most important pathway in the tissue destruction associated with periodontal disease [7]. And based on previous studies, dramatically elevated levels of MMP-1, MMP-2, MMP-3, MMP-8, and MMP-9 have been detected in gingival crevicular fluid, peri-implant sulcular fluid, and gingival tissue of periodontitis patients [8]. Likewise, recent studies have also shown that mRNA levels of MMPs are significantly increased in inflamed gingival tissue. MMPs activity may be regulated by interactions with their endogenous inhibitors (TIMPs) and posttranslational modifications, as well as at the levels of gene transcription [9]. Consequently, it can be hypothesized that functional polymorphisms in MMPs genes may affect MMPs expression or activity and, thus, may predispose to periodontal disease conditions.

According to some genotype analyses of single nucleotide polymorphisms (SNPs) in MMPs genes, they have shown increased frequency of several common MMPs SNPs in patients with periodontitis [10–13]. On the contrary, some other studies have demonstrated little or no association of these SNPs in MMPs genes with etiopathogenesis of periodontitis [14–17]. Despite comprehensive studies focusing on the association of gene polymorphisms with the susceptibility and/or severity of periodontitis, there exists a high degree of inconsistency and the results are inconclusive; therefore, for the purpose of deriving a more precise estimation of association between these MMPs SNPs and periodontitis risk, we performed a meta-analysis and systematic review of all eligible studies.

## 2. Materials and Methods

### 2.1. Protocols and Eligibility Criteria

The meta-analysis and systematic review reported here are in accordance with the Preferred Reporting Items for Systematic Review and Meta-Analyses (PRISMA) statement (Appendix S1 in the Supplementary Material available online at http://dx.doi.org/10.1155/2016/1545974). The research question for this study was formulated based on the PICO (population, intervention, comparison, and outcomes) criteria. The literature search was limited to original studies performed in humans on the association of matrix metalloproteinases SNPs with periodontitis risk.

### 2.2. Search Strategy

Studies addressing the correlations of MMPs genetic polymorphisms with the risk of periodontitis were identified by performing an electronic search in PubMed (1966 to May 2015), Medline (1950 to May 2015), and Web of Science databases (1900 to May 2015) by using the following search terms in PubMed: (((((((“Matrix Metalloproteinases” [Mesh]) OR Matrix Metalloproteinases) OR Matrix Metalloproteinase) OR MMPs) OR MMP)) AND (((((“Polymorphism, Genetic” [Mesh]) OR Polymorphism) OR “Genetic Variation” [Mesh]) OR Genetic Variation) OR genetic variant)) AND (((((((((((“Periodontitis” [Mesh]) OR Periodontitis) OR “Chronic Periodontitis” [Mesh]) OR Chronic Periodontitis) OR CP) OR “Aggressive Periodontitis” [Mesh]) OR Aggressive Periodontitis) OR AgP) OR “Periodontal Diseases” [Mesh]) OR Periodontal Diseases) OR PD). Other databases were searched with comparable terms suitable for the specific database. Furthermore, in order to identify any additional studies that may have been missed, a computer-assisted strategy based on manual searching of reference lists from potentially relevant reviews and retrieved articles was performed. Full texts of the relevant articles and studies published in English were retrieved and included to explore the association between MMPs polymorphisms and the susceptibility to periodontitis.

### 2.3. Selection of Studies

The studies included in the present meta-analysis and systematic review had to meet the following inclusion criteria: (a) studies used validated genotyping methods (such as PCR-RFLP and TaqMan) to measure the association of SNPs in MMP genes with periodontitis risk; (b) studies were in an appropriate analytical design, including case-control, cohort, or nested case-control; (c) studies were published in English; (d) the full text of studies was available, and (e) the data of studies were not duplicated in another manuscript. However, studies were excluded if they did not provide enough information on genotype frequency or did not report sufficient genotype distribution for calculation of odds ratios (ORs) and its variance. Besides, studies investigating the mixed population were excluded if they did not provide the detailed information for each ethnicity. Moreover, studies were also excluded if genotype distributions of control subjects were varied from Hardy-Weinberg equilibrium (HWE).

### 2.4. Data Extraction

To ensure homogeneity of data collection and to rule out the effect of subjectivity in data gathering, data extraction was performed independently by two investigators (Ying Zhu and Pradeep Singh), using a predefined protocol. Disagreements were resolved by iteration, discussion, and consensus. A series of items were collected for each trial, including first author's surname, publication year, country, ethnicity (Caucasian, Asian, or mixed (excluding the detailed ethnic results of mixed population in the original study)), type and severity of periodontitis, matching criteria of cases and controls, source of controls, allelic frequency in both cases and controls, genotyping methods, and also the genes and variants genotyped. Furthermore, the evidence of HWE in controls was verified through the application of an online software (http://www.oege.org/software/hwe-mr-calc.shtml). *p* value less than 0.05 of HWE was considered to be significant.

### 2.5. Risk of Bias

Methodological quality was independently evaluated by two researchers (Pradeep Singh and Deepal Haresh Ajmera) according to the recently proposed Newcastle-Ottawa Scale (NOS) criteria for the quality assessment of case-control studies. To unravel potential systematic biases, a third investigator (Wenyang Li) performed a concordance study by independently reviewing all eligible studies; complete concordance was reached for all variables assessed. Briefly, the quality of each study was assessed by using the following methodological components: (1) subject selection; (2) comparability of subject; and (3) clinical outcome.  Table 2 illustrates the details of each methodological item. NOS scores ranged from 0 to 9, wherein a score of ≥5 was regarded as high-quality study, while studies with scores <5 were classified as low-quality studies.

### 2.6. Heterogeneity

A test for heterogeneity (true variance of effect size across studies) was performed using a *Q* test (to assess whether observed variance exceeds expected variance) to establish inconsistency in the study results. However, because the test is susceptible to the number of trials included in the meta-analysis, we also calculated *I* ^2^. *I* ^2^, directly calculated from the *Q* statistic, indicates the percentage of variability in effect estimates because of true heterogeneity, rather than sampling error. *I* ^2^ ranges from 0% to 100%, with 0% indicating the absence of any heterogeneity. Although absolute numbers for *I* ^2^ are not available, values <50% are considered low heterogeneity, and the effect is thought to be fixed. Conversely, when *I* ^2^ exceeds 50%, then heterogeneity is thought to exist and the effect is random.

### 2.7. Statistical Analysis

The STATA version 11.0 (Stata Corp, College Station, TX, USA) software was used for meta-analysis. The strength of the association between MMPs SNPs and periodontitis risk was evaluated by ORs with their 95% confidence intervals (CIs) under different genetic models: the allele model (mutant allele versus wild allele), the codominant model (homozygous rare/heterozygous versus homozygous frequent and homozygous rare versus heterozygous), the dominant model (heterozygous + homozygous rare versus homozygous frequent), and the recessive model (homozygous rare versus heterozygous + homozygous frequent), as well as the additive model (heterozygous versus homozygous frequent + homozygous rare). In addition, subgroup analyses were stratified, when feasible, according to the type of disease, racial descent, severity of chronic periodontitis, and smoking habit, respectively. The *Z*-test was used to estimate the statistical significance of pooled ORs, and the Bonferroni correction was used to account for multiple testing in association analyses. When all genetic models were tested for each SNP, a corrected *p* value < 0.01 was considered statistically significant.

To estimate the pooled ORs, a fixed effects model (the Mantel-Haenszel method) was used initially, whereas the random effects model (DerSimonian and Laird method) was applied when evidence of significant heterogeneity was found across trials (*p* < 0.1 and *I* ^2^ > 50%). In order to evaluate the potential source of heterogeneity, a sensitivity analysis was performed through sequential removal of each included study. Publication bias was investigated using funnel plots, wherein the standard error of log(OR) was plotted against log(OR) for each study. Besides, funnel plot asymmetry was assessed with the Begg rank correlation test (Begg test) and the Egger linear regression approach (Egger test). *p* values of less than 0.05 from the Egger's test were considered statistically significant. In addition, the results of the trials which could not be pooled through the meta-analysis were assessed using descriptive statistics.

## 3. Results

**Figure 1:**
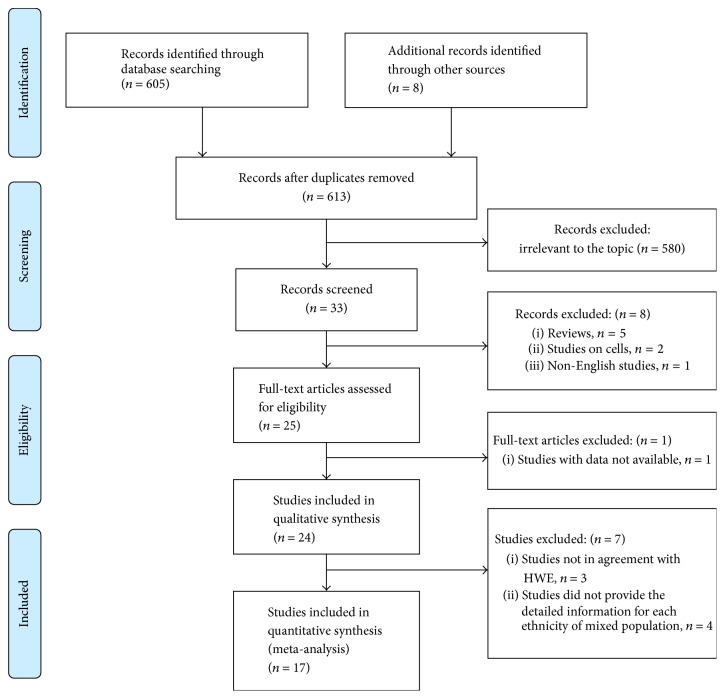
Flow of study identification, inclusion, and exclusion.

The flowchart for the process of including/excluding articles is shown in Figure 1. After abstracts were screened for relevance, 25 full-text studies, comprising chronic periodontitis (CP) and/or aggressive periodontitis (AgP), were comprehensively assessed against the inclusion criteria. Three studies were excluded because they were not in accordance with HWE [10, 18, 19]. Another four studies were excluded because they reported the results of mixed population but did not provide the detailed information for each ethnicity [15, 17, 20, 21]. Besides, one more study was excluded due to insufficient data availability for calculating ORs and their variance [7]. Finally, 17 case-control studies, investigating the association of* MMP-1* (−1607 1G/2G, −519 A/G, and −422 A/T),* MMP-2* (−1575 G/A, −1306 C/T, −790 T/G, and −735 C/T),* MMP-3* (−1171 5A/6A),* MMP-8* (−799 C/T, −381 A/G, and +17 C/G),* MMP-9* (−1562 C/T and +279 R/Q),* MMP-12* (−357 Asn/Ser), and* MMP-13* (−77 A/G and 11A/12A) with periodontitis risk, were included in this meta-analysis [8, 11–14, 16, 22–32]. And the characteristics and quality assessment of all included studies are summarized in Tables 1 and 2.

### 3.1. MMP-1

**Figure 2:**
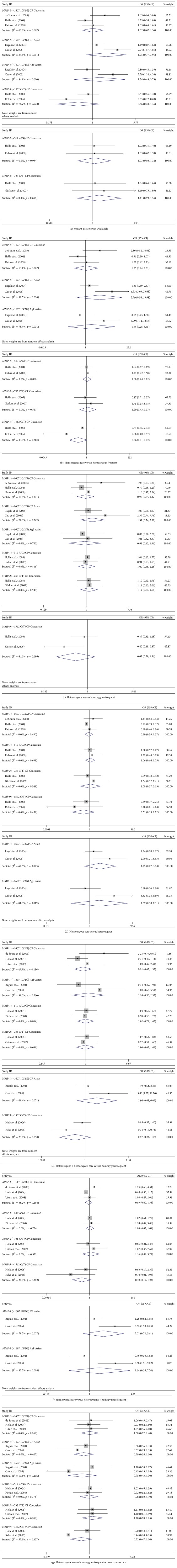
Forest plot of periodontitis risk associated with MMPs polymorphisms under all comparison models.

Table 3 and Figure 2 show the meta-analysis results of two SNPs in the* MMP-1* gene, namely, −1607 1G/2G and −519 A/G, under various genetic models. In Caucasians, we failed to identify any significant association of these two SNPs with the susceptibility to CP under all comparison models (Table 3; Figure 2). Besides, in Asian population, our results also demonstrated that there was no statistically significant association between* MMP-1* −1607 1G/2G polymorphism and the risk of both CP and AgP (Table 3; Figure 2). Furthermore, analyses of individual polymorphism revealed no differences in distribution of* MMP-1* −422 A/T variant between CP and control groups in Caucasians [14].

Considering the influence of disease severity on polymorphism, we also performed stratified analysis by severity of CP. Pooled ORs revealed that no significant association existed between* MMP-1 *−1607 1G/2G polymorphism and the risk of mild to moderate or severe CP in both Caucasians and Asians under all comparison models (Table 3). Besides, a study by Pirhan et al. [26] reported that the −519 G allele carrying genotypes of* MMP-1* gene was not suggested to be related with severe CP in Caucasian population (adjusted OR = 1.25, *p* = 0.83). With smoking being one of the major contributing factors in the susceptibility of periodontitis, we also performed subgroup analysis according to the smoking habit of subjects. In Caucasians, our results revealed that when only nonsmoking or smoking subjects were included, the difference between* MMP-1* −1607 1G/2G polymorphism in CP patients and control population was not significant under all comparison models (Table 3). Likewise, apparent association could not be related with the stratified analysis by individual smoking habit in the allelic and genotype frequencies of MMP-1 −519 A/G polymorphism between CP and control groups in Caucasian population [14]. On the contrary, the results by Holla et al. [14] also suggested that there were significant differences in the distribution of* MMP-1* −422 A/T variant between a subgroup of smoking CP patients versus smoking controls in Caucasians (*p* = 0.017).

### 3.2. MMP-2

In the present meta-analysis, we failed to associate* MMP-2* −735 C/T polymorphism with CP risk in Caucasian population under all comparison models (Table 3; Figure 2). Besides, a study by Gürkan et al. [8] revealed that this SNP was also not related to AgP risk in Caucasians. Similarly, no significant association of* MMP-2* −1575 G/A, −1306 C/T, and −790 T/G SNPs with the susceptibility to periodontitis was observed in Caucasian and Asian populations [16, 23].

As far as the severity of CP was considered, the allelic and genotype distributions of* MMP-2* −735 C/T variant were similar in severe CP and healthy subjects in Caucasians [25]. When stratified by smoking habit, we found that this polymorphism was not linked with the risk of CP in nonsmoking Caucasian patients and controls without smoking history under all comparison models (Table 3). Likewise, the results of subgroup analysis by Gürkan et al. [8] showed that there was no significant difference regarding the distribution of this SNP between nonsmoking AgP and nonsmoking healthy subjects in Caucasian population. Besides, a similar distribution of other three* MMP-2* variants was also observed between CP patients and controls in subgroup analysis according to smoking status in Caucasians [23].

### 3.3. MMP-9

Our meta-analysis results revealed that* MMP-9* −1562 C/T SNP might not contribute to CP risk in Caucasians under all comparison models (Table 3; Figure 2). Likewise, the results by Chen et al. [16] and Gürkan et al. [8] failed to find a significant association of this variant with the risk of AgP in Asian and Caucasian populations, respectively. Besides, any significant association of* MMP-9* +279 R/Q polymorphism with the susceptibility to CP was also absent in Caucasians [24].

When stratified by the severity of CP, pooled ORs also did not reveal any significant association between* MMP-9* −1562 C/T variant and severe CP risk in Caucasians under all comparison models (Table 3). Similarly, it was reported by Holla et al. [24] that there was no difference in the distribution of* MMP-9* +279 R/Q SNP between the Caucasian CP patients with mild to moderate disease and those with severe disease. Concerning the smoking habit of subjects, the results by Holla et al. [24] suggested no significant difference in the allele and genotype frequencies of* MMP-9* −1562 C/T polymorphism between smoking or nonsmoking CP patients and controls with or without smoking history in Caucasians. Moreover, when the smokers were excluded, the distribution of this SNP in the nonsmoking Caucasian subjects with AgP was similar to that in the healthy group [8].

### 3.4. Other MMPs

One SNP, −1171 5A/6A (in the promoter region of* MMP-3* gene), has been investigated. In the study by Itagaki et al. [22], they failed to support the influence of this polymorphism on susceptibility to both CP (*p* = 0.935) and AgP (*p* = 0.057) in Asians. Moreover, as far as the severity of CP was concerned, the results by Itagaki et al. [22] also revealed that in Asian population, there were no statistically significant differences in the distribution of this variant among three CP phenotypes (severe, moderate, and slight) (*p* = 0.240, 0.188, and 0.114, resp.).

Variation in* MMP-8* gene, particularly of −799 C/T, −381 A/G, and +17 C/G SNPs, has been investigated in association with periodontitis. As for* MMP-8* −799 C/T polymorphism, analysis of genotypes in periodontitis and healthy control groups in the study by Chou et al. [11] showed that the −799 T allele was associated with increased risk of both AgP (adjusted OR = 1.99, *p* = 0.04) and CP (adjusted OR = 1.93, *p* = 0.007) in Asians. Likewise, Emingil et al. [30] has also found analogous results for the association between this variant and AgP risk (*p* < 0.005) in Caucasians. On the contrary, the results by Holla et al. [29] suggested no differences in the allelic and genotype frequencies of this SNP between Caucasian CP patients and controls (*p* > 0.05). Besides, as for* MMP-8* −381 A/G and +17 C/G polymorphisms, studies conducted by Holla et al. [29] and Emingil et al. [30] revealed that there was no significant association of these two SNPs with the susceptibility to periodontitis in Caucasians. When stratified by smoking habit, a significant difference in T allele carriers of* MMP-8* −799 C/T polymorphism in both AgP (adjusted OR = 2.33, *p* < 0.05) and CP (adjusted OR = 1.84, *p* < 0.05) groups versus control group was found in nonsmokers subgroup analysis in Asian population [11], while studies by Holla et al. [29] and Emingil et al. [30] showed no association of all these three* MMP-8* variants with the risk of CP and AgP in Caucasians when the group of subjects was divided according to smoking status.

A few articles have reported the relation of* MMP-12* −357 Asn/Ser as well as* MMP-13* −77 A/G and 11A/12A SNPs to periodontitis risk in Caucasian population. In the studies by Gürkan et al. [8, 25], they could not succeed in establishing the relationship of* MMP-12* −357 Asn/Ser variant with the susceptibility either to AgP (OR = 1.29, 95% CI = 0.64–2.61; *p* = 0.47) or to severe CP (OR = 0.80, 95% CI = 0.31–2.03; *p* = 0.56). Similarly, a study conducted by Pirhan et al. [28] also failed to reveal any significant influence regarding the distribution of* MMP-13* −77 A/G (OR = 0.11, 95% CI = 0.01–1.59; *p* = 0.11) and 11A/12A (data not shown, *p* > 0.05) polymorphisms on severe CP risk. Furthermore, in the nonsmoker subgroup analysis, the allelic and genotype frequencies of* MMP-12* −357 Asn/Ser variant in the nonsmoking subjects with AgP or CP was similar to those in the healthy group according to studies by Gürkan et al. [8, 25].

### 3.5. Publication Bias and Sensitivity Analysis

The results of these two analyses are shown in Appendices S2 and S3.

## 4. Discussion

*MMP-1* −1607 1G/2G, located on 11q22-q23 chromosome, is one of the most studied SNPs in periodontitis. Evidence from previous studies revealed that individuals carrying 2G/2G genotype appeared to be at greater risk for developing periodontitis than individuals who had 1G/1G and 1G/2G genotypes [26, 32]. Although the exact mechanism behind these findings is not known, it has reported that the presence of 2G allele together with an adjacent adenosine creates a core binding site (5′-GGA-3′), which is the consensus sequence for the Ets family of transcription factors immediately adjacent to an AP-1 site [33]. Moreover, carriage of 2G allele is also shown to augment transcriptional activity by 37-fold and may potentially increase the levels of protein expression [34]. This mechanism provides the molecular bases for a more intense degradation of periodontal extracellular matrix, leading to increased risk of periodontitis.

However, in our study we could not only find any significant association between* MMP-1* −1607 1G/2G polymorphism and periodontitis risk, but also failed to associate* MMP-1* −519 A/G and −422 A/T SNPs with the susceptibility to periodontitis. Several reasons may contribute to our results. First, an overview of clinical outcomes revealed that, even with the same genotype, the presence of a high variation in MMP-1 expression among periodontitis individuals could be due to additional influence of specific periodontopathogens and cytokine stimulation [35]. Based on the results of these studies, a stronger signaling because of intense and sustained stimulation of host cells by periodontopathogens and by the inflammatory mediators (such as IL-1b and TNF-a) characteristically induced by them may overcome the genetic predisposition, and high levels of* MMP-1* are transcribed irrespective of these SNPs [36].

Also, it is believed that the combination of several significant gene variants in certain individuals synergistically elevate the susceptibility to disease [37]. Since role of TIMPs in MMPs function cannot be denied, it can also be postulated that mutation of the position 2 (Thr in TIMP-1) greatly affects the affinity of TIMPs for MMPs and substitution to glycine essentially inactivates TIMP-1 for MMPs inhibition [38], thus potentiating MMPs activity. Besides, results of the linkage disequilibrium and the haplotype frequencies of* MMP-1* and* MMP-3* variants, both of which are located in 11q22.3 chromosome near to each other, indicated that the risk 2G allele in* MMP-1* was more frequently linked to the nonrisk 6A allele in* MMP-3*, suggesting that the risk and nonrisk linkage combination might lead to the functional compensation of MMP function, to put it in another way, protective function of host homeostasis [22].

Furthermore, previous studies have hypothesized that covariates like severity of the disease and smoking may contribute towards regulation of MMP-1 expression in diseased periodontium [39]. So, we also performed subgroup analyses according to severity of CP and smoking habit of subjects. Similarly, lack of association between* MMP-1* gene variants in terms of CP severity as well as smoking status and periodontitis risk was observed in the present meta-analysis and systematic review. All these above results may lead to the conclusion that an increase in mRNA transcription caused by these* MMP-1* promoter SNPs may not necessarily lead to an increased effect of MMP-1 on the extracellular matrix of periodontal tissues, and many other factors such as bacterial metabolites, cytokines, and other gene variants are supposed to be involved in the regulation of MMP-1 expression and functionality.

The* MMP-2* −735 C/T polymorphism is a synonymous mutation, resulting in the same amino acid (threonine) at codon 460 regardless of the allele present. It has been shown that variation of this SNP at synonymous sites could lead to allele-specific structural differences in mRNA that could affect mRNA structure dependent mechanisms [40], which could have functional consequences of increased MMP-2 expression. In oral cancer, previous studies have verified that patients with* MMP-2* −735 CC genotype present increased risk for developing oral squamous cell carcinoma when compared to those with CT or TT genotype [41]. These findings were consistent with other studies that have linked this genotype with an increased risk of development of lung cancer [42], gastric cardia adenocarcinoma [43], and abdominal aortic aneurysm [44], suggesting that this polymorphism is identified as a promising candidate for neoplasms.

On the contrary, several studies failed to show association between this variant and the susceptibility to periodontitis [8, 23, 25]. Likewise, our results also found no association of this SNP with the risk of both CP and AgP, so did* MMP-2* −1575 G/A and −1306 C/T, as well as −790 T/G SNPs. A possible explanation would be that the rare allele of these variants could disrupt a Sp-1 binding site within the promoter region of* MMP-2* gene, thus leading to lower* MMP-2* promoter activity [45], which might also contribute towards negative association of these* MMP-2* polymorphisms with periodontitis risk. Besides, when stratified by the severity of CP and smoking, a similar distribution of all these* MMP-2* variants was observed between periodontitis patients and controls. So, we can make a conclusion that genetically determined mechanisms may not be important in tuning the effect of MMP-2 on periodontal tissues.

*MMP-9* −1562 C/T SNP, located on 20q11.2-q13.1 chromosome, has been under investigation for its association with an increased risk for the development of cancer and emphysema as well as many other diseases [46]. Based on the evidence of previous studies, the suggested mechanism behind a positive association of this polymorphism with disease risk might be that the MMP-9 expression is primarily controlled at the transcriptional level, where the promoter of* MMP-9* gene responds to stimuli of various cytokines and growth factors [47]. Furthermore, the T allele of this variant can abolish a binding site for a transcription repressor and, thus, change the promoter activity of* MMP-9*, leading to increased MMP-9 expression. Besides, an exchange of C-to-T at position −1562 can also alter the binding of a nuclear protein to this region, resulting in increased transcriptional activity in macrophages [48].

However, the present study failed to find any association of both* MMP-9* −1562 C/T and +279 R/Q SNPs with periodontitis risk. A possible explanation for this discrepancy may be that not only the variant, but several binding sites and also their length-dependent interaction with nuclear proteins may influence the transcriptional activity of the gene due to its close localization to the transcriptional start site [49]. In addition, recent evidence indicates that, in periodontitis, changes in MMP/TIMP balance occur as a result of physiological ageing and that gender might be a significant factor modifying this balance [50]. Although multiple genetic factors, including SNPs, are involved in pro- and anti-inflammatory situations, effect of other factors like oxidant-antioxidant imbalance and tissue remodeling cannot be denied and should be simultaneously considered to understand the entire picture of periodontitis risk.

The −1171 5A/6A variant, a well-characterized insertion/deletion polymorphism in the promoter region of* MMP-3* gene, is considered to be functionally involved in the process of periodontitis. The 5A allele of this polymorphism has been shown to result in higher MMP-3 expression and enzyme activity, thereby increasing extracellular matrix breakdown because of disruption of a binding site for a nuclear factor kappa B, which acts as a transcriptional repressor [51]. Moreover, some studies reported positive association of this SNP with periodontitis and concluded that individuals with the 5A/5A genotype were 2-3 times more likely to develop periodontitis [15, 19]. Conversely, several other studies showed a nonsignificant trend for association of this variant with periodontitis, suggesting a likely attempt of the host environment to contain and perhaps specifically outbalance the increased MMP-3 levels to minimize tissue damage [7, 22].

Recently, several studies have investigated* MMP-8* −799 C/T, −381 A/G, and +17 C/G variants in different periodontal diseases. However, a significant correlation with periodontitis risk was only found in* MMP-8* −799 C/T polymorphism, and it has been reported that T allele carriers have more MMP-8 production in the periodontal environment with bacterial challenge compared to non-T allele carriers [30]. The exact mechanism behind this association is still unknown, but T allele of this variant has been proved to have about 1.8-fold higher promoter activity than the C allele [52]. Besides, MMP-8 activity has also been found to be modified in various organs and body fluids in smokers [53, 54], and tobacco-induced degranulation events in neutrophils and increase in proinflammatory mediator burden can influence the expression level of MMP-8 in smokers' periodontal environment [55]. However, none of the previous studies have succeed in associating these* MMP-8* variants with smoking and periodontitis risk, indicating smoking status may not exert an effect on the association of these SNPs with periodontitis susceptibility.

*MMP-12* −357 Asn/Ser as well as* MMP-13 *−77 A/G and 11A/12A SNPs, located on 11q22.2-q22.3 chromosomes, has been evaluated with the periodontitis risk in a limited number of studies. And it is suggested that all these polymorphisms do not appear to have a significant influence on the susceptibility to periodontitis and are also not associated with the clinical severity of periodontitis as well as outcome of periodontal therapy and gingival crevicular fluid MMP-12/-13 levels [28]. Moreover, recent studies have also revealed that* MMP-2*,* MMP-3*,* MMP-7*,* MMP-8*,* MMP-11*, or* MMP-12* single gene knockout mice failed to show any apparent disorders, suggesting that a single SNP of MMP might not contribute enough in the susceptibility or progression of a disease. A likely explanation for this behavior would be the sharing of common extracellular matrix substrates by some MMP members which might even compensate these functions for each other [56]. Furthermore, lack of association between these variants and periodontitis may also suggest that an increase in the MMP-12 or MMP-13 transcriptions may not necessarily lead to an increase in the destructive effect of these enzymes on the periodontal tissues.

When compared with previous similar meta-analysis and systematic reviews [57, 58], the present study has several strengths. First, almost all of these prior studies pooled ORs by using the data of trials investigating the mixed population; however, a meta-analysis of mixed ethnicities is meaningless for a genetic association study, owing to high population heterogeneity. As a result, we excluded the trials if they did not provide the detailed information for each ethnicity of a mixed population; moreover, in order to get more reliable results, all meta-analyses and subgroup analyses in our study were performed according to the racial descent. Besides, some of the previous meta-analyses even included studies in which genotype distributions of control subjects were varied from HWE; however, the allele-frequency comparison test is valid only if HWE conditions prevail. Therefore, in the current study, we also took into consideration this factor that might bias the results, suggesting that evidence from our meta-analysis should be considered to be convincing. Nevertheless, this study still has several potential limitations. One potential limitation is that our restriction on searching studies published in indexed journals and also studies published only in English could introduce an inherent bias for this analysis. Moreover, lack of information for the adjustments of major confounders including age, gender, and environmental factors might cause confounding bias so a more precise analysis would have been performed if all individual raw data had been available. Finally, there were only two ethnicity groups (Caucasian and Asian) included in the present study. Thus, it is doubtful whether the obtained conclusions were generalizable to other populations. Further studies on this topic in different ethnicities are expected to be conducted to strengthen our results.

In conclusion, the present meta-analysis and systematic review suggested that although studies of the association between* MMP-8* −799 C/T variant and the susceptibility to periodontitis have not yielded consistent results,* MMP-1* (−1607 1G/2G, −519 A/G, and −422 A/T),* MMP-2* (−1575 G/A, −1306 C/T, −790 T/G, and −735 C/T),* MMP-3* (−1171 5A/6A),* MMP-8* (−381 A/G and +17 C/G),* MMP-9* (−1562 C/T and +279 R/Q), and* MMP-12* (−357 Asn/Ser), as well as* MMP-13* (−77 A/G and 11A/12A) SNPs are not related to periodontitis risk. However, further well-designed studies with larger sample size and more ethnic groups are required to validate the negative association identified in our study. Besides, we expect that in the future, analyses using polymorphisms will not only identify individual variations within disease comparisons but also help in identification of human response to various therapies. Consequently, even though significant insights have been gained into the role of MMPs and their function, a lot of work needs to be done before the roles of MMPs in development of periodontitis are fully elucidated.

## Supplementary Material

Appendix S1: The Preferred Reporting Items for Systematic Review and Meta-Analyses (PRISMA) statement of our study.
Appendix S2: The results of publication bias.
Appendix S3: The results of sensitivity analysis.

## Figures and Tables

**Table 1:** Main Characteristics of included studies.

Author, year	Country	Ethnicity	Sample size (case/control)	Type of periodontitis	Matching criteria	Genotype method	Gene (polymorphism) & HWE in controls
de Souza et al., 2003 [31]	Brazil	Caucasian	50/37	CP (moderate or severe)	Smoker ratios	PCR-RFLP	MMP-1 (−1607 1G/2G) 0.87

Holla et al., 2004 [14]	Czech Republic	Caucasian	133/196	CP (mild to moderate to severe)	Age, gender	PCR-RFLP	MMP-1 (−1607 1G/2G) 0.52 MMP-1 (−519 A/G) 0.11 MMP-1 (−422 A/T) 0.47

Itagaki et al., 2004 [22]	Japan	Asian	205/142	CP (mild or moderate or severe)	Age, gender, smoker ratios	TaqMan	MMP-1 (−1607 1G/2G) 0.48 MMP-3 (−1171 5A/6A) 0.87
			37/142	AgP			

Holla et al., 2005 [23]	Czech Republic	Caucasian	149/127	CP (mild to moderate to severe)	Age, smoker ratios	PCR-RFLP	MMP-2 (−1575 G/A) 0.40 MMP-2 (−1306 C/T) 0.19 MMP-2 (−790 T/G) 0.67 MMP-2 (−735 C/T) 0.42

Cao et al., 2005 [32]	China	Asian	40/52	AgP	—	PCR-RFLP	MMP-1 (−1607 1G/2G) 0.78

Cao et al., 2006 [13]	China	Asian	60/50	CP (moderate or severe)	—	PCR-RFLP	MMP-1 (−1607 1G/2G) 0.99

Keles et al., 2006 [12]	Turkey	Caucasian	70/70	CP (severe)	Age, gender	PCR-RFLP	MMP-9 (−1562 C/T) 0.82

Holla et al., 2006 [24]	Czech Republic	Caucasian	169/135	CP (moderate or severe)	Age, gender, smoker ratios	PCR-RFLP	MMP-9 (−1562 C/T) 0.59 MMP-9 (+279 R/Q) 0.25

Chen et al., 2007 [16]	China	Asian	79/128	AgP	Age, gender	DHPLC PCR-RFLP	MMP-2 (−1306 C/T) 1.00 MMP-9 (−1562 C/T) 0.63

Gürkan et al., 2007 [8]	Turkey	Caucasian	92/157	AgP	Gender	PCR-RFLP	MMP-2 (−735 C/T) 0.45 MMP-9 (−1562 C/T) 0.35 MMP-12 (357 Asn/Ser) 0.06

Gürkan et al., 2008 [25]	Turkey	Caucasian	87/107	CP (severe)	—	PCR-RFLP	MMP-2 (−735 C/T) 0.43 MMP-12 (357 Asn/Ser) 0.47

Pirhan et al., 2008 [26]	Turkey	Caucasian	102/98	CP (severe)	—	PCR-RFLP	MMP-1 (−519 A/G) 0.79

Ustun et al., 2008 [27]	Turkey	Caucasian	126/54	CP (moderate or severe)	Age	PCR-RFLP	MMP-1 (−1607 1G/2G) 0.75

Pirhan et al., 2009 [28]	Turkey	Caucasian	102/98	CP (severe)	—	PCR-RFLP	MMP-13 (−77 A/G) 0.89 MMP-13 (11A/12A) 0.92

Chou et al., 2011 [11]	China	Asian	361/106	CP (moderate to severe)	Gender, smoker ratios	PCR-RFLP	MMP-8 (−799 C/T) 0.22
			96/106	AgP			

Holla et al., 2012 [29]	Czech Republic	Caucasian	341/278	CP (mild to moderate to severe)	Age, gender, smoker ratios	PCR-RFLP	MMP-8 (+17 C/G) 0.14 MMP-8 (−799 C/T) 0.63

Emingil et al., 2014 [30]	Turkey	Caucasian	100/167	AgP	Gender	PCR-RFLP	MMP-8 (+17 C/G) 0.29 MMP-8 (−799 C/T) 0.09 MMP-8 (−381 A/G) 0.87

CP: chronic periodontitis; AgP: aggressive periodontitis; HWE: Hardy-Weinberg equilibrium. Mild chronic periodontitis: patients with teeth exhibiting < 3 mm attachment loss; moderate chronic periodontitis: patients with teeth exhibiting ≥ 3 mm and <7 mm attachment loss; severe chronic periodontitis: patients with teeth exhibiting ≥ 7 mm attachment loss. A *p* value less than 0.05 of HWE was considered significant.

**Table 2:** Assessing the quality of included studies.

Author, year	Selection				Comparability		Exposure			Score
de Souza et al., 2003 [31]	☆			☆	☆		☆	☆		5

Holla et al., 2004 [14]	☆		☆	☆	☆	☆	☆	☆		7

Itagaki et al., 2004 [22]	☆			☆	☆	☆	☆	☆		6

Holla et al., 2005 [23]	☆		☆	☆	☆	☆	☆	☆		7

Cao et al., 2005 [32]	☆			☆	☆		☆	☆		5

Cao et al., 2006 [13]	☆			☆	☆		☆	☆		5

Keles et al., 2006 [12]	☆	☆	☆	☆	☆		☆	☆		7

Holla et al., 2006 [24]	☆	☆	☆	☆	☆	☆	☆☆	☆		9

Chen et al., 2007 [16]	☆		☆	☆	☆	☆	☆☆	☆		8

Gürkan et al., 2007 [8]	☆	☆		☆	☆		☆☆	☆		7

Gürkan et al., 2008 [25]	☆	☆		☆	☆		☆☆	☆		7

Pirhan et al., 2008 [26]	☆	☆		☆	☆	☆	☆	☆	☆	8

Ustun et al., 2008 [27]	☆			☆	☆		☆	☆		5

Pirhan et al., 2009 [28]	☆	☆		☆	☆		☆☆	☆		7

Chou et al., 2011 [11]	☆	☆		☆	☆	☆	☆	☆		7

Holla et al., 2012 [29]	☆	☆	☆	☆	☆	☆	☆	☆		8

Emingil et al., 2014 [30]	☆	☆		☆	☆	☆	☆	☆		7

Selection	(1) Is the case definition adequate?									
	(a) Yes, with independent validation ☆									
	(b) Yes, for example, record linkage or based on self-reports									
	(c) No description									
	(2) Representativeness of the cases									
	(a) Consecutive or obviously representative series of cases ☆									
	(b) Potential for selection biases or not stated									
	(3) Selection of controls									
	(a) Community controls ☆									
	(b) Hospital controls									
	(c) No description									
	(4) Definition of controls									
	(a) No history of disease (endpoint) ☆									
	(b) No description of source									

Comparability	(1) Comparability of cases and controls on the basis of the design or analysis									
	(a) Study controls for the most important factor (HWE in control group) ☆									
	(b) Study controls for any additional factor (e.g., age, gender, and smoker ratios) ☆									

Exposure	(1) Ascertainment of exposure									
	(a) Secure record ☆									
	(b) Structured interview where blind to case/control status ☆									
	(c) Interview not blinded to case/control status									
	(d) Written self-report or medical record only									
	(e) No description									
	(2) Same method of ascertainment for cases and controls									
	(a) Yes ☆									
	(b) No									
	(3) Nonresponse rate									
	(a) Same rate for both groups ☆									
	(b) Nonrespondents described									
	(c) Rate different and no designation									

**Table 3:** Meta-analysis results of the polymorphisms in MMPs gene on periodontitis risk.

MMP-1								
−1607 1G/2G	Studies (cases/controls)	2G versus 1G OR (95% CI) *I* ^2^ (%), *p*_*h*_, *p*_*c*_	2G/2G versus 1G/1G OR (95% CI) *I* ^2^ (%), *p*_*h*_, *p*_*c*_	1G/2G versus 1G/1G OR (95% CI) *I* ^2^ (%), *p*_*h*_, *p*_*c*_	2G/2G versus 1G/2G OR (95% CI) *I* ^2^ (%), *p*_*h*_, *p*_*c*_	1G/2G + 2G/2G versus 1G/1G OR (95% CI) *I* ^2^ (%), *p*_*h*_, *p*_*c*_	2G/2G versus 1G/1G + 1G/2G OR (95% CI) *I* ^2^ (%), *p*_*h*_, *p*_*c*_	1G/2G versus 1G/1G + 2G/2G OR (95% CI) *I* ^2^ (%), *p*_*h*_, *p*_*c*_

Type of disease								
*CP*								
Caucasian	3 (309/287)	1.02 (0.67–1.56)	1.05 (0.44–2.51)	0.95 (0.64–1.42)	0.90 (0.59–1.37)	0.91 (0.62–1.32)	0.89 (0.60–1.33)	1.00 (0.72–1.40)
		63.1, 0.067, **1.000**	63.0, 0.067, **1.000**	12.0, 0.321, **1.000**	0.0, 0.490, **1.000**	49.9, 0.136, **1.000**	38.2, 0.198, **1.000**	0.0, 0.969, **1.000**
Asian	2 (302/192)	1.75 (0.77–3.95)	2.79 (0.56–13.98)	1.31 (0.74–2.32)	1.75 (0.77–3.94)	1.96 (0.63–6.09)	2.01 (0.72–5.61)	0.79 (0.55–1.16)
		84.5, 0.011, **1.000**	81.5, 0.020, **1.000**	27.0, 0.242, **1.000**	64.6, 0.093, **1.000**	69.4, 0.071, **1.000**	79.7, 0.027, **1.000**	0.0, 0.467, **1.000**
*AgP*								
Asian	2 (77/194)	1.34 (0.48–3.73)	1.54 (0.28–8.55)	0.91 (0.42–1.96)	1.67 (0.38–7.31)	1.14 (0.56–2.32)	1.64 (0.35–7.70)	0.75 (0.43–1.30)
		84.8, 0.010, **1.000**	78.4, 0.031, **1.000**	0.0, 0.763, **1.000**	81.8, 0.019, **1.000**	39.0, 0.200, **1.000**	85.7, 0.008, **1.000**	59.5, 0.116, **1.000**

Severe CP								
*Caucasian*								
Mild to moderate	2 (66/91)	0.99 (0.63–1.55)	0.99 (0.39–2.50)	1.16 (0.52–2.60)	0.85 (0.39–1.84)	1.10 (0.51–2.38)	0.89 (0.43–1.85)	1.17 (0.62–2.21)
		0.0, 0.613, **1.000**	0.0, 0.613, **1.000**	0.0, 0.667, **1.000**	0.0, 0.874, **1.000**	0.0, 0.613, **1.000**	0.0, 0.751, **1.000**	0.0, 0.876, **1.000**
Severe	2 (110/91)	1.53 (0.72–3.24)	2.44 (0.47–12.59)	1.52 (0.70–3.30)	1.31 (0.68–2.51)	1.68 (0.81–3.50)	1.47 (0.80–2.73)	0.98 (0.56–1.73)
		65.7, 0.088, **1.000**	63.4, 0.098, **1.000**	15.8, 0.276, **1.000**	0.0, 0.369, **1.000**	51.1, 0.153, **1.000**	42.3, 0.188, **1.000**	0.0, 0.845, **1.000**
*Asian*								
Mild to moderate	2 (168/192)	1.26 (0.93–1.72)	1.62 (0.84–3.12)	1.40 (0.72–2.69)	1.19 (0.75–1.87)	1.51 (0.82–2.80)	1.27 (0.83–1.95)	0.97 (0.63–1.48)
		60.1, 0.113, **0.987**	62.7, 0.101, **1.000**	43.8, 0.182, **1.000**	0.0, 0.534, **1.000**	56.0, 0.132, **1.000**	21.0, 0.261, **1.000**	0.0, 0.784, **1.000**
Severe	2 (97/192)	1.99 (0.92–4.26)	2.93 (0.71–12.03)	1.16 (0.53–2.56)	2.19 (1.26–3.79)	1.78 (0.86–3.67)	2.55 (0.95–6.85)	0.58 (0.35–0.98)
		73.5, 0.052, **0.546**	67.0, 0.082, **0.952**	0.0, 0.468, **1.000**	48.9, 0.162, **0.035**	38.1, 0.204, **0.854**	69.7, 0.069, **0.441**	0.0, 0.517, **0.287**

Smoking habit in CP								
*Caucasian*								
Nonsmoking	3 (200/213)	0.92 (0.55–1.55)	0.90 (0.33–2.43)	0.87 (0.54–1.40)	0.85 (0.52–1.41)	0.96 (0.45–2.05)	0.82 (0.52–1.30)	0.98 (0.66–1.46)
		66.1, 0.053, **1.000**	63.1, 0.067, **1.000**	34.9, 0.215, **1.000**	0.0, 0.438, **1.000**	56.9, 0.098, **1.000**	40.1, 0.118, **1.000**	0.0, 0.575, **1.000**
Smoking	2 (109/74)	1.10 (0.71–1.72)	1.14 (0.43–3.02)	1.12 (0.54–2.34)	1.15 (0.50–2.64)	1.12 (0.55–2.29)	1.19 (0.53–2.65)	0.98 (0.53–1.84)
		19.0, 0.267, **1.000**	32.9, 0.222, **1.000**	0.0, 0.976, **1.000**	52.2, 0.148, **1.000**	0.0, 0.682, **1.000**	54.0, 0.140, **1.000**	0.0, 0.319, **1.000**

MMP-1								
−519 A/G	Studies (cases/controls)	G versus A OR (95% CI) *I* ^2^ (%), *p*_*h*_, *p*_*c*_	GG versus AA OR (95% CI) *I* ^2^ (%), *p*_*h*_, *p*_*c*_	AG versus AA OR (95% CI) *I* ^2^ (%), *p*_*h*_, *p*_*c*_	GG versus AG OR (95% CI) *I* ^2^ (%), *p*_*h*_, *p*_*c*_	AG + GG versus AA OR (95% CI) *I* ^2^ (%), *p*_*h*_, *p*_*c*_	GG versus AA + AG OR (95% CI) *I* ^2^ (%), *p*_*h*_, *p*_*c*_	AG versus AA + GG OR (95% CI) *I* ^2^ (%), *p*_*h*_, *p*_*c*_

Type of disease								
*CP*								
Caucasian	2 (235/293)	1.03 (0.80–1.32)	1.08 (0.64–1.82)	1.00 (0.68–1.46)	1.06 (0.64–1.75)	1.02 (0.71–1.45)	1.06 (0.67–1.69)	0.98 (0.69–1.39)
		0.0%, 0.984, **1.000**	0.0%, 0.806, **1.000**	0.0%, 0.811, **1.000**	0.0%, 0.691, **1.000**	0.0%, 0.884, **1.000**	0.0%, 0.736, **1.000**	0.0, 0.778, **1.000**

MMP-2								
−735 C/T	Studies (cases/controls)	T versus C OR (95% CI) *I* ^2^ (%), *p*_*h*_, *p*_*c*_	TT versus CC OR (95% CI) *I* ^2^ (%), *p*_*h*_, *p*_*c*_	CT versus CC OR (95% CI) *I* ^2^ (%), *p*_*h*_, *p*_*c*_	TT versus CT OR (95% CI) *I* ^2^ (%), *p*_*h*_, *p*_*c*_	CT + TT versus CC OR (95% CI) *I* ^2^ (%), *p*_*h*_, *p*_*c*_	TT versus CC + CT OR (95% CI) *I* ^2^ (%), *p*_*h*_, *p*_*c*_	CT versus CC + TT OR (95% CI) *I* ^2^ (%), *p*_*h*_, *p*_*c*_

Type of disease								
*CP*								
Caucasian	2 (236/234)	1.11 (0.79–1.55)	1.19 (0.43–3.37)	1.12 (0.74–1.68)	1.08 (0.37–3.13)	1.00 (0.67–1.49)	1.16 (0.41–3.24)	1.10 (0.74–1.65)
		0.0, 0.695, **1.000**	0.0, 0.511, **1.000**	0.0, 0.940, **1.000**	0.0, 0.541, **1.000**	0.0, 0.699, **1.000**	0.0, 0.522, **1.000**	0.0, 0.989, **1.000**

Smoking habit in CP								
*Caucasian*								
Nonsmoking	2 (133/198)	1.13 (0.74–1.72)	1.15 (0.32–4.09)	1.17 (0.70–1.94)	0.99 (0.27–3.66)	1.16 (0.71–1.88)	1.10 (0.31–3.89)	1.15 (0.69–1.90)
		0.0, 0.337, **1.000**	45.2, 0.177, **1.000**	0.0, 0.784, **1.000**	32.4, 0.224, **1.000**	0.0, 0.533, **1.000**	42.4, 0.188, **1.000**	0.0, 0.917, **1.000**

MMP-9								
−1562 C/T	Studies (cases/controls)	T versus C OR (95% CI) *I* ^2^ (%), *p*_*h*_, *p*_*c*_	TT versus CC OR (95% CI) *I* ^2^ (%), *p*_*h*_, *p*_*c*_	CT versus CC OR (95% CI) *I* ^2^ (%), *p*_*h*_, *p*_*c*_	TT versus CT OR (95% CI) *I* ^2^ (%), *p*_*h*_, *p*_*c*_	CT + TT versus CC OR (95% CI) *I* ^2^ (%), *p*_*h*_, *p*_*c*_	TT versus CC + CT OR (95% CI) *I* ^2^ (%), *p*_*h*_, *p*_*c*_	CT versus CC + TT OR (95% CI) *I* ^2^ (%), *p*_*h*_, *p*_*c*_

Type of disease								
*CP*								
Caucasian	2 (239/205)	0.56 (0.24–1.35)	0.36 (0.11–1.12)	0.63 (0.29–1.36)	0.51 (0.15–1.72)	0.57 (0.23–1.38)	0.39 (0.12–1.24)	0.72 (0.47–1.10)
		78.2, 0.032, **1.000**	35.9, 0.212, **0.553**	64.0, 0.096, **1.000**	0.0, 0.459, **1.000**	73.9, 0.050, **1.000**	20.4, 0.262, **0.777**	57.1, 0.127, **0.924**

Severe CP								
*Caucasian*								
Severe	2 (163/205)	0.63 (0.20–1.97)	0.44 (0.13–1.49)	0.71 (0.24–2.09)	0.53 (0.14–1.95)	0.65 (0.19–2.15)	0.46 (0.14–1.57)	0.75 (0.28–2.01)
		86.1, 0.007, **1.000**	54.4, 0.139, **1.000**	79.3, 0.028, **1.000**	0.0, 0.428, **1.000**	84.2, 0.012, **1.000**	41.0, 0.193, **1.000**	76.0, 0.041, **1.000**

MMP-1: matrix metalloproteinase-1; MMP-2: matrix metalloproteinase-2; MMP-9: matrix metalloproteinase-9; CP: chronic periodontitis; AgP: aggressive generalized periodontitis.

*p*_*h*_: the *p* value of heterogeneity; *p*_*c*_: the *p* value corrected by Bonferroni correction; OR: odds ratio; CI: confidence interval.

When *p*_*h*_ is <0.1 and *I* ^2^ exceeds 50%, the random effects model is used. Conversely, the fixed effects model is used.

*p*_*c*_ < 0.01 is considered statistically significant.
