# Supplementary material for: Association of Common Variants in MMPs with Periodontitis Risk
Source: Dis Markers. 2016 Apr 19;2016:1545974. doi: 10.1155/2016/1545974 (PMC4853955; doi:10.1155/2016/1545974)
Supplement: Supplementary file 1 — Appendix S1: The Preferred Reporting Items for Systematic Review and Meta-Analyses (PRISMA) statement of our study. Appendix S2: The results of publication bias. Appendix S3: The results of sensitivity analysis. [file 1545974.f1.zip › Suppl/Appendix S2.doc]

**Publication Bias**

Because the number of included trials for each meta-analysis is relatively small, a funnel plot for pooled estimates to assess the potential publication bias has not been performed. As a result, there may be a publication bias as well, which could result in the inaccurate estimation of the association between these MMPs SNPs and periodontitis risk.

**Sensitivity analysis**

As for *MMP-1* -1607 1G/2G variant, we performed disease-specific and smoking-specific sensitivity analyses. Our results revealed that in Caucasian population, the study conducted by de Souza et. al. [22] was the main contributor of heterogeneity in subgroups of chronic periodontitis and non-smoking subjects. The exclusion of this study could eliminate the heterogeneity, and using a fixed-effects model, pooled ORs in subgroup of CP still reached the same results (Appendix S3). In subgroup of non-smoking subjects, with the study by de Souza et. al. [22] excluded, pooled ORs of the remaining trials in a fixed-effects model still got the same results under co-dominant (2G/2G vs. 1G/1G) and dominant (2G/2G+1G/2G vs. 1G/1G) models (Appendix S3); however, after excluding the same study, we found that there was a significant difference regarding the allele frequency of *MMP-1* -1607 1G/2G polymorphism between non-smoking CP patients and non-smoking controls in Caucasian population under the allele model (OR=0.71, 95% CI=0.51–0.98, P=0.037), with no evidence of heterogeneity across the remaining trials (P_h_=0.732, *I^2^*=0.0%) (Appendix S3).
