# Supplementary material for: Association of Common Variants in MMPs with Periodontitis Risk
Source: Dis Markers. 2016 Apr 19;2016:1545974. doi: 10.1155/2016/1545974 (PMC4853955; doi:10.1155/2016/1545974)
Supplement: Supplementary file 1 — Appendix S1: The Preferred Reporting Items for Systematic Review and Meta-Analyses (PRISMA) statement of our study. Appendix S2: The results of publication bias. Appendix S3: The results of sensitivity analysis. [file 1545974.f1.zip › Suppl/Appendix S3.doc]

| **Appendix S3: Sensitivity analysis for** **heterogeneity** | | | | | |
| --- | --- | --- | --- | --- | --- |
| **MMP-1**  **(-1607 1G/2G)** | **Study majorly contributed to**  **the heterogeneity** | **Heterogeneity** **after excluding the study** | | | **Pooled ORs of the remaining studies** |
|  |  | **P_h_** | ***I^2^*,%** | | **OR 95% CI, *P*** |
| ***Type of disease*** | | | | | |
| **CP** | | | | | |
| **Caucasian** | | | | | |
| 2G vs. 1G | de Souza et al.’s study | 0.261 | | 20.8 | 0.83(0.64, 1.08), *0.159* |
| 2G/2G vs. 1G/1G | de Souza et al.’s study | 0.268 | | 18.5 | 0.69(0.41, 1.17), *0.168* |
| ***Smoking habit in CP*** | | | | | |
| **Caucasian** | | | | | |
| **Non-smoking** | | | | | |
| **2G vs. 1G** | **de Souza et al.’s study** | **0.732** | | **0.0** | **0.71(0.51, 0.98), *0.037*** |
| 2G/2G vs. 1G/1G | de Souza et al.’s study | 0.786 | | 0.0 | 0.54(0.29, 1.03), *0.060* |
| Dominant genetic model | de Souza et al.’s study | 0.526 | | 0.0 | 0.66(0.40, 1.07), *0.094* |
| MMP-1: matrix metalloproteinase-1; CP: chronic periodontitis; Dominant genetic model: 2G/2G+1G/2G vs. 1G/1G;  P_h_: the P-value of heterogeneity; OR: odds ratio; CI: confidence interval. | | | | | |
